# Teacher-made models: the answer for medical skills training in developing countries?

**DOI:** 10.1186/1472-6920-12-98

**Published:** 2012-10-19

**Authors:** Trung Q Tran, Albert Scherpbier, Jan Van Dalen, Pamela E Wright

**Affiliations:** 1 University of Medicine and Pharmacy, Ho Chi Minh, Vietnam; 2 Maastricht University, Maastricht, the Netherlands; 3 Medical Committee Netherlands-Vietnam, Amsterdam, the Netherlands

**Keywords:** Clinical skills laboratory, Teacher made models, Commercial models, Vietnam

## Abstract

**Background:**

The advantages of using simulators in skills training are generally recognized, but simulators are often too expensive for medical schools in developing countries. Cheaper locally-made models (or part-task trainers) could be the answer, especially when teachers are involved in design and production (teacher-made models, TM).

**Methods:**

We evaluated the effectiveness of a TM in training and assessing intravenous injection skills in comparison to an available commercial model (CM) in a randomized, blind, pretest-posttest study with 144 undergraduate nursing students. All students were assessed on both the TM and the CM in the pre-test and post-test. After the post-test the students were also assessed while performing the skill on real patients.

**Results:**

Differences in the mean scores pre- and post-test were marked in all groups. Training with TM or CM improved student scores substantially but there was no significant difference in mean scores whether students had practiced on TM or CM. Students who practiced on TM performed better on communication with the patient than did students who practiced on CM. Decreasing the ratio of students per TM model helped to increase practice opportunities but did not improve student’s mean scores. The result of the assessment on both the TM and the CM had a low correlation with the results of the assessment on real persons.

**Conclusions:**

The TM appears to be an effective alternative to CM for training students on basic IV skills, as students showed similar increases in performance scores after training on models that cost considerably less than commercially available models. These models could be produced using locally available materials in most countries, including those with limited resources to invest in medical education and skills laboratories.

## Background

Medical simulators are revolutionizing training for the practice of medicine [1-5]. Currently [2] thousands of schools around the world are using simulators for hands-on health care education to medical, nursing, and allied health students. Skills laboratories (skillslabs) have been successfully developed in resource-rich countries [1,6-10]. These skillslabs vary in the accommodation provided and the resources available from one room with one manikin to purpose-built structures with a vast assortment of equipment [6,7,11,12]. In Japan, students must pass the Common Achievement Test, which includes an objective structured clinical examination, before starting their clinical education. Many medical schools have been under pressure to provide clinical skills laboratories for their students. A range of simulators are used in skills laboratories [7].

Using simulation, medical students can safely learn, practice, and repeat a skill or procedure over and over until proficiency is achieved, without touching a real patient. They can improve their skills and confidence without compromising patient safety [6,7,11,12]. But it is not always appropriate to transfer this model to medical and healthcare schools in developing countries, because the capital and maintenance costs may far exceed local budgets [13]. The equipment and manikins are very expensive, especially the more sophisticated types. Japanese medical colleges invested up to 600,000 USD for the necessary equipment of a clinical skills laboratory [7]. Once set up and equipped, the maintenance and running cost may prove prohibitive in the long term, so that the equipment cannot be repaired or maintained [13]. Moreover, even the expensive models have limited validity. For example, they do not help to prepare students for communication with their patients during procedures.

In Vietnam, we have obtained financial support from the Dutch Government and World Bank for setting up skills laboratories in most of the medical schools, but the sustainability of these skills laboratories is still a challenge. The school fee is only about 300 USD per year per student, and university funds are insufficient to buy more simulation models to replace broken ones. One solution for sustainability is to make the models ourselves. We established a unit for learning material research and development, whose main role is to motivate and support teachers to design and produce learning materials (including skills training models, or part-task trainers) using appropriate techniques and locally available resources. There are only teachers and support staff in the unit; no “special experts” are involved.

One of the very first teacher made models was a simple model for training and assessing intravenous (IV) injection procedure. We started with this model because intravenous injection is an invasive procedure and the commercial model soon breaks down. Moreover, we could make this model simply and cheaply (5 USD compared to 300–400 USD for a commercial IV model) and it will last longer because of its structure. These teacher-made models (TM) helped us not only in replacing the broken commercial models but also in increasing the number of models available for our students to use for practice in their training sessions. Custer et al. found that increasing the number of performances tended to increase the quality and speed of the performance [14]. By supplying more models, we expected that our students would have more opportunities for practicing and that the quality of skills training would therefore improve.

Furthermore, clinical skills include more than just the procedure. Patient-based simulation has enormous potential as learning tool, and can provide insight into the subtleties and complexities that characterize clinical practice [2]. Because our TM for IV injection is attached to a simulated patient’s arm, we hoped that it could also help students learn to communicate better with the patient.

Even an inexpensive model will be too expensive and wasteful if it does not function well in training and assessment. Therefore, we investigated whether there were differences in effectiveness of using teacher made models compared to the commercially available models in our university in training and assessing students on the skill ‘intravenous injection’.

Sub question 1: Do students reach similar levels of technical skill when trained on TM compared to CM?

Sub question 2: Do students communicate better with patients when trained on TM compared to CM?

Sub question 3: Do students reach a higher level of technical skills level when trained more often with TM?

To answer these questions, we conducted an experimental study to compare the results of skills assessment on three groups of students who practiced with CM, with TM or with an increased number of TM.

## Methods

### Setting

This study was conducted at the Faculty of Nursing and Medical Technology, University of Medicine and Pharmacy at Ho Chi Minh City (UMP HCMC), the largest health university in Vietnam. Before practicing the skill on models, all students had studied the related theory. Normally one teacher and one teaching assistant train 20–25 students during 50 minute sessions working with three models for intravenous injection in the arm. The Commercial Models (CM) used in this study were new plastic arms for intravenous injection training available at our university (Intravenous Training Arm, 300–400 USD, supplied by Gaumard Scientific Company, USA).

The teacher made model (TM) or part-task trainer used in this study was developed by teachers of the Skills Lab at UMP HCMC and had been tested and improved over a period of time. The TM for IV injection is a hand-made silicone bandage which has one vein, a thin protection under the vein and a blood container. The vein can be seen and palpated on the silicone ‘skin’ surface. The model is wrapped around the forearm of a simulated patient (SP) for practicing IV placement, injection of fluids, and drawing of blood (See also Additional file 1.). In this study, students following the same course in IV injection played the role of simulated patients (SP). No extra training was given to these peer SP because the scenario was very simple: the student SP only had to respond to the greeting (if any) and give their name. The students practicing had to check whether they had the right client then inform and explain the steps of the procedure to the patient.

Based on our experience with the TM, our hypothesis was that there would be no difference in effectiveness between using TM and CM in training and assessment of intravenous injection practice. With the advantages of very low cost and making them ourselves, we could supply more TM for the students, thus giving students more opportunities for practice and hopefully leading to better performance. Because the TM was attached to a person, we expected that students would learn better to communicate with the patient during training on TM compared to the separate inanimate arm of the CM.

### Research design

The study was set up to compare the effectiveness of the teacher made model (TM) with the available commercial model (CM) in two different training situations: using the same ratio of students per model and using different ratios of students per model. We tried to make the research fit within the routine training and assessment program.**Participants:**The participants in this study were the students and teachers who normally participate in training and assessing these skills. They were not recruited especially for the study; instead the study was carried out during the normal training program. The student participants were all of the 144 first year students in the primary program of nursing who were going to be trained in IV procedure. The students were divided into three groups in the order of their normal classes; the groups were comparable in age, gender and learning achievements. All of the students learned the theory together, and were trained in the skills laboratory using the same lesson plan. The same group of teachers and teaching conditions and the same group of raters for all assessments were applied under the same conditions. The only difference in the training of the three groups was the simulation model:

Group 1 used the available commercial model with the ratio of 8 students per model;

Group 2 used the teacher made model with the ratio of 8 students per model;

Group 3 used the teacher made model with the ratio of 2 students per model.

### Three-step assessment process

Students were assessed three times in the training process, using the same assessment procedure and rating scale each time. The reliability and validity of the tools had been evaluated. Pre-practice assessment was designed to provide a “baseline” of the student’s competency before practicing on the models and to check if the IV injection procedure really needs to be trained using a model. Post- practice assessment served to check direct effects of training on the model and assessment with real patient was to check the effect on practice in the real clinical situation.

#### Pre-practice assessment

Before practicing on models, students were assessed on performance of the IV procedure on the model they were going to practice with (group 1 with CM, groups 2 and 3 with TM).

#### Post-practice assessment

After practicing on models, students were assessed on their performance of the IV procedure on two different kinds of model, first on the model they had used in practice (group 1 with CM, groups 2 and 3 with TM) and later, on the other model. Because each assessment could also be considered as a practice opportunity, we designed the study such that students were assessed with the model used in practice first and then with the other model, giving the TM and CM the same opportunity to contribute to skill-building during assessment.

#### Assessment with real patient

When the students went to the hospital for practicing on real patients, they were evaluated while performing the intravenous injection on their first real patient, under supervision of the teacher.

### Objective assessment

All of the assessments used the same assessment tools, which had been validated in national assessments. The raters (observers) were trained until the inter-rater reliability reached 0.8**.** Raters were the same for all groups of students and were blinded: the raters did not know which students had belonged to which study group. The rating scale had five points (0= does not do, 1= Poor; 2= Fair; 3= Good; 4= Excellent) and included 15 items related to the IV procedure and 1 item on interaction. The maximum score for the 15 items was 60. (The rating scale is attached as Additional file 2.)

The teachers who developed the TM did not participate in the student assessment, and the teachers who did student assessment did not know on which models the students had been trained. Although bias because of teachers wishing to promote the TM would have been possible it was avoided in this study (Table 1).

**Table 1:** Summary of the research design

**Group**	**Model used in training**	**Models used in assessment**		
		**Pre-practice assessment**	**Post-practice assessment**	**First real patient assessment**
1	8 students/CM	Commercial model	commercial model then teacher-made model	real patient
2	8 students/TM	teacher-made model	teacher-made then commercial model	real patient
3	2 students/TM	teacher-made model	teacher-made then commercial model	real patient

### Ethical approval

Ethical approval was not required for this study, which was carried out as part of the regular training of the students in the University. However, approval of the project including ethical aspects was provided by the Ho Chi Minh City University of Medicine and Pharmacy. In Vietnam, after training in a skillslab, students practice in teaching hospitals under supervision by teachers. The students tested for this study were carrying out their normal practice supervised by teachers. The only difference was that the teachers marked the students with the same rating scale as used in the skillslab. Before practice on patients, students asked patients’ consent verbally. When patients come to teaching hospitals, they normally agree to allow students to practice, under supervision by the teachers.

### Statistical analysis

Data analysis was performed using SPSS 13.0. One-way ANOVA and paired-samples tests were used. The level of significance for all comparisons was set at P < 0.05. In the tables showing results, the significant differences are marked and the 95% confidence intervals given.

## Results

### Results of pre-practice assessment

One way-ANOVA analysis of the results of the pre-practice assessment showed no significant differences in performance scores among the students in the three groups (See also Additional file 3).

### Results of post-practice assessment using CM

A paired-samples test comparing the results of the post-practice assessment using CM with pre-practice assessment showed that the performance of the students in all three groups increased significantly in all items of the IV procedure after the described practice sessions.

There was no significant difference in the mean score of students in group 1 (trained on CM) compared with groups 2 and 3 (trained on TM). Comparing groups 2 and 3, only for item 4 (Select the right injection site) was the mean score of group 3 (practiced more often on TM) significantly higher than that of group 2 (practiced less often on TM) (See also Additional file 4).

Groups 1, 2 and 3 were trained and assessed in the same conditions, except for the number of models used in practice sessions. Table 2 shows that by decreasing the student/model ratio from 8 to 2, the mean number of practice times did differ significantly (Table 2).

**Table 2:** Number of times practiced on model

**GROUP**		**N**	**Minimum**	**Maximum**	**Mean**	**Std. Deviation**
1	8 Students/1 CM	49	1	2	1.14	.35
2	8 Students/1 TM	48	1	2	1.04	.20
3	2 Students/1 TM	47	2	6	3.77*	1.10

* Significantly different from groups 1 and 2, p<0.05.

### Results of post-practice assessment using TM

The results of the post-practice assessment using TM showed no significant differences in mean scores of students in groups 1 and 2. Comparing groups 1 and 3, the only significant difference was in item 3 (Check the right client with the physician’s order, prepare patient) where group 3 performed better than group 1. Comparing groups 2 and 3, only for item 2 (Prepare medication) was the mean score of students in group 2 significantly higher than in group 3 (See also Additional file 5).

### Results of assessment of performance on real patients

Assessment of students’ performance when carrying out the IV procedures on their first real patient revealed no significant differences in mean scores in most the items of the IV procedure. The exceptions were for item 3 (Check the right client with the physician’s order, prepare patient) where groups 2 and 3 performed better than group 1 and for item 12 (Check the right position of the needle) where group 3 performed better than group 2 (See also Additional file 6).

### Result of the assessment using TM, CM or real persons

For the TM, the correlation between performance scores on models and on real patients was only significant for 10 of the 15 items, and the correlation for these items was low (ranging from .175 to .341). For the CM, the correlation was significant in 13 of the 15 items; correlation of these items was also low (from .176 to .466).

### Differences of the three groups in assessment results

**Figure 1:**
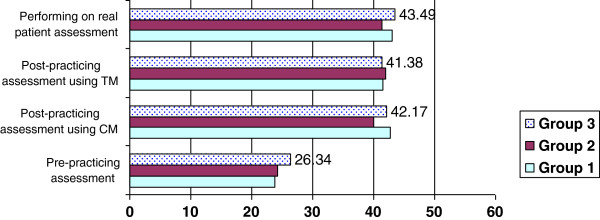
Means of the total scores in the three assessments of 3 groups.

Figure 1: Means of the total scores in the three assessments of 3 groups, where 60 is the maximum score (4/4 points on 15 items).

In Figure 1, the results are summarized. There was no significant difference in mean scores between students who practiced on TM (group 2) and students who practiced on CM (group 1) in the post-practice assessment. That result was the same whether CM or TM was used in a post-practice assessment. In assessment of the skill performance on a real patient, the results were quite comparable. Only on Item 3 (Check the right client with the physician’s order, prepare patient) which is related to communication with the patient, did groups who had practiced on TM (groups 2 and 3) perform better than group 1, who practiced only on CM.

## Discussion

The results of this study suggest that TM were at least equal to CM in providing the opportunity for students to acquire the expected IV procedure skills. The cost of CM models for schools in developing countries may be too high; the initial investment may already far exceed local budgets, and maintenance is equally important [13]. Students who practiced on the TM model outperformed their classmates who practiced on the CM on one key item related to communication skills. The TM was bandaged on the arm of a simulated patient, which encourages the student to pay attention to the person, not only the arm, and provides opportunity for the communication with the patient. This result fits with the recent attention to the benefits of attaching models to human beings to provide a more realistic experience while learning technical skills [15,16].

In Group 3, we tried to maximize the number of TM for students. As a result, the number of times that students practiced the skill increased significantly. This implies that if students are given more practice opportunities they will use them. However, the results of their assessments were not significantly different in most of the items. This result is not consistent with the common idea of “practice makes perfect” nor with the results reported by Custers et al. [14], suggesting that increasing the number of times of practice will increase students’ competence. We provided enough models so that instead of eight students per model we had only two. That meant that one student had the model bandaged to his arm while the other performed the procedure. In each of these training sessions there were 24 students with one teacher, one teaching assistant and twelve (instead of three) models. That meant that twelve students practiced simultaneously, making good supervision and feedback by the two teachers more difficult. In addition, when one student wears the model and one practices, there are no other students to use the observer checklists and provide peer feedback. If the students in Group 3 made errors, were not corrected and repeated them through practice, they may have become proficient not only in good practice but also in bad practice [17]. This is an undesirable side-effect of the teacher-student ratio. A ratio of 3 or 4 students/model might have been better, to provide peer observers during practice. Another explanation could be that although group 3 students had more chance to practice, students in groups 1 and 2 had more chances to observe which is also a good way to learn [14]. The best balance between observation and practice and therefore the optimal student/model ratio for each training condition should be investigated in further research.

The low correlation between the results of assessments on models and on real persons could arise from the differences between models (TM or CM) and real persons, but it could also arise from the limited stability of the students’ performance. When we validate the result of an assessment by comparing it with the result of another assessment, we assume that the competence shown by the students will be consistent. To validate these comparisons, it would be useful to study different subjects with different levels of competence.

The relatively small sample size is a limitation of this study, and we cannot be sure that the results on this teacher-made model will apply to other TM. We have not yet investigated what the students thought and felt when learning using the different models. There is scope for further research into these questions.

## Conclusions

The TM appears to be an effective and appropriate alternative to CM for training on basic IV skills, as students showed a similar increase in performance while being trained on models that cost considerably less than the commercially available models. This is especially important in settings with limited resources such as developing countries but may also be useful in developed countries. Because this TM is attached to a simulated patient, it may provide an extra benefit, increasing the learning on communication with patients while carrying out the procedure. Increasing the number of models without changing other aspects of the training setting did not improve the results of student assessment. The effectiveness of using TM or CM in assessment for predicting the students’ performance on real patients remains a question.

## Abbreviations

TM: Teacher made model; CM: Commercial model; IV: Intravenous injection.

## Competing interests

Although there is potential for competing interests in the sense that the first author has developed models that could be produced and sold commercially, in fact because we are providing sufficient information for anyone in another location to make their own models, we feel there is no competing interest. We have not registered this model for any kind of copyright or patent. We encourage medical schools in limited resource settings to explore making their own models from locally available resources.

## Authors’ contributions

TQ Tran designed and implemented the study, analyzed the data and drafted the manuscript. A Scherpbier contributed to formulation of the study questions and study design and advised on the preparation of the manuscript. J van Dalen advised on the study questions and study design and contributed to the preparation of the manuscript. EP Wright advised on processing, selection and interpretation of research results and formulation of the paper, shared in writing and editing the manuscript. All authors have read and approved the final version of the manuscript.

## Pre-publication history

The pre-publication history for this paper can be accessed here:

http://www.biomedcentral.com/1472-6920/12/98/prepub

## Supplementary Material

Additional file 1 Teacher made model for IV procedures. Click here for file

Additional file 2 Rating scale for intravenous injection. Click here for file

Additional file 3 Compare the results of the pre-practice assessment. Click here for file

Additional file 4 Results of post-practice assessment using CM. Click here for file

Additional file 5 Results of post-practice assessment using TM. Click here for file

Additional file 6 Results of assessment of performance on real patients. Click here for file
