# Supplementary material for: Teacher-made models: the answer for medical skills training in developing countries?
Source: BMC Med Educ. 2012 Oct 19;12:98. doi: 10.1186/1472-6920-12-98 (PMC3533861; doi:10.1186/1472-6920-12-98)
Supplement: Additional file 1 — Teacher made model for IV procedures. [file 1472-6920-12-98-S1.doc]

**Appendix 1: Teacher made model for IV procedures**


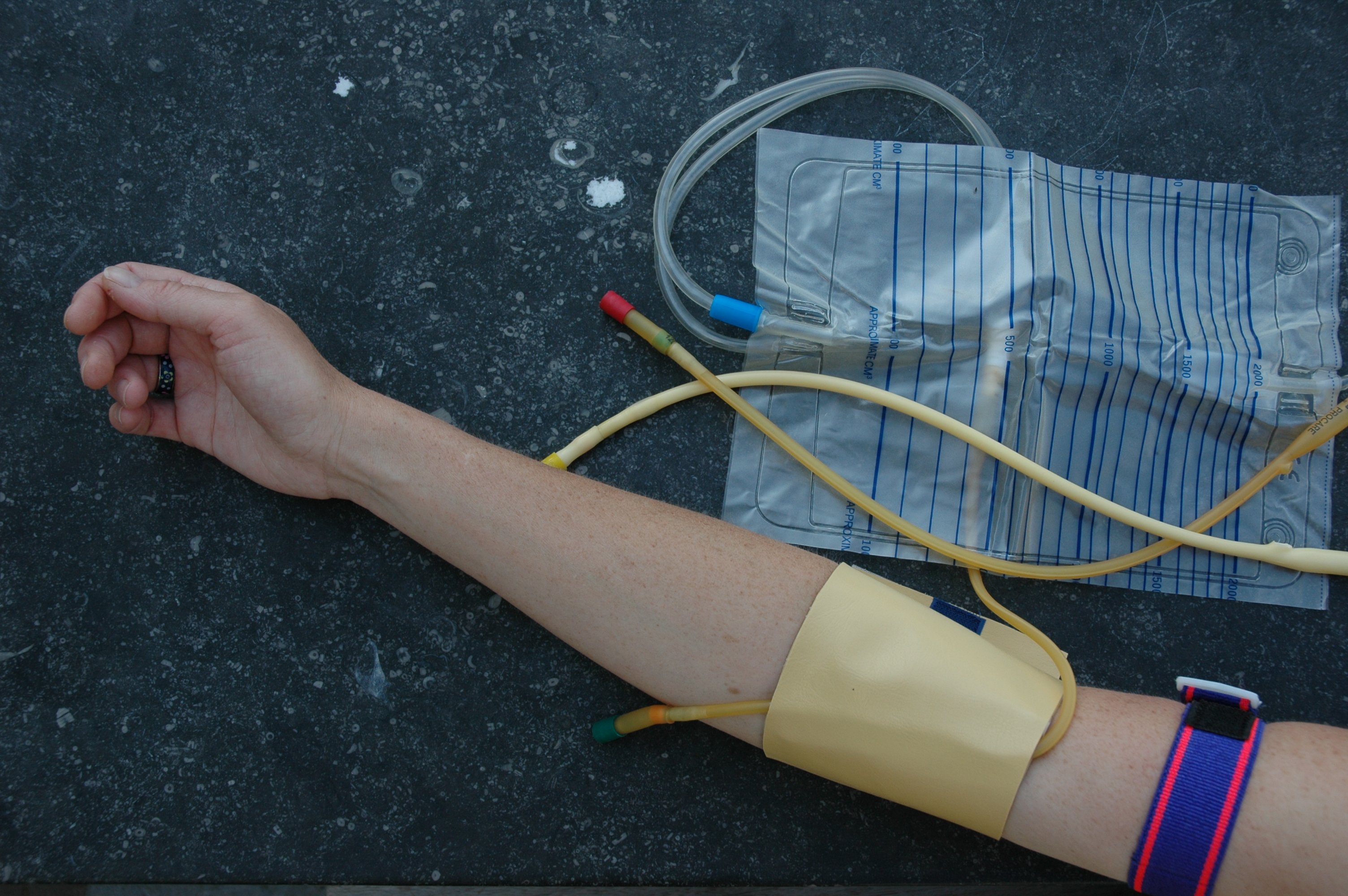


***Teacher made model (TM):***

Used in practice of IV placement, injection of fluids, and drawing of blood.

***Structure:***

One vein in a hand-made silicone bandage; thin protection under the vein; blood container is modified from a urine bag.

***Features***

- Easy to use

- The vein can be seen and palpated on the skin surface

- Enables communication with the patient (TM is bandaged on simulated patient’s arm)

- Damaged part of the vein can be cut out and the rest of the vein be reused

- In our setting, the cost for making model is around 5 USD, in which material costs 3 USD
