# Supplementary material for: Teacher-made models: the answer for medical skills training in developing countries?
Source: BMC Med Educ. 2012 Oct 19;12:98. doi: 10.1186/1472-6920-12-98 (PMC3533861; doi:10.1186/1472-6920-12-98)
Supplement: Additional file 2 — Rating scale for intravenous injection. [file 1472-6920-12-98-S2.doc]

**Appendix 2:** Rating scale for intravenous injection

| No | **CONTENTS** | **0** | **1** | **2** | **3** | **4** |
| --- | --- | --- | --- | --- | --- | --- |
| **1** | **Check right medication, wear mask and wash your hands.** |  |  |  |  |  |
|  | Prepare medication card. |  |  |  |  |  |
|  | Check medication order. |  |  |  |  |  |
|  | Wear mask and wash your hand | 0 |  | 2 |  | 4 |
| **2** | **Prepare medication** |  |  |  |  |  |
|  | Sterilize top of ampoule with alcohol swab, check medication (2^nd^ time). |  |  |  |  |  |
|  | Snap neck of ampoule. |  |  |  |  |  |
|  | Draw up medication safely. |  |  |  |  |  |
|  | Checking medication (3^rd^ time), discard the ampoule in medical waste container. |  |  |  |  |  |
|  | Cap the needle on the syringe (or replace other needle) putting safety syringe and needle in a tray. |  |  |  |  |  |
| **3** | **Check the right client with the physician’s order, prepare patient:** |  |  |  |  |  |
|  | Check that you have the right client with the physician’s order. |  |  |  |  |  |
|  | Inform and explain steps of procedure to patient |  |  |  |  |  |
| **4** | **Select appropriate injection site** |  |  |  |  |  |
|  | Expose the injection site. | 0 |  | 2 |  | 4 |
|  | Select appropriate injection site. |  |  |  |  |  |
| **5** | **Cleanse the entry site:** |  |  |  |  |  |
|  | Apply disposable gloves. | 0 |  |  |  | 4 |
|  | Apply flat tourniquet around arm, 10 to 15 cm above proposed injection site. |  |  |  |  |  |
|  | Cleanse injection site with alcohol swab (middle to outward). |  |  |  |  |  |
| **6** | **Wash hands again with disinfection solution or alcohol swab.** | 0 |  | 2 |  | 4 |
| **7** | **Remove air bubbles in syringe.** |  |  |  |  |  |
| **8** | **Ensure that the bevel side of the needle is facing up.** | 0 |  |  |  | 4 |
| **9** | **Stretch the skin against the direction of insertion to the site.** | 0 |  | 2 |  | 4 |
| **10** | **Insert at 30-40 degree angle.** | 0 |  | 2 |  | 4 |
| **11** | **Advance the needle into the vein.** |  |  |  |  |  |
| **12** | **Check the right position of the needle.** |  |  |  |  |  |
| **13** | **Aspirate by pulling back gently on the plunger of syringe to determine the needle is in a blood vessel, release the tourniquet.** | 0 |  |  |  | 4 |
| **14** | **Inject the medication into the vein.** |  |  |  |  |  |
|  | Inject the medication slowly, monitor patient’s response carefully. |  |  |  |  |  |
|  | Withdraw the needle quickly at the same angle at which it was inserted, placing the alcohol swab on the injection site. |  |  |  |  |  |
| **15** | **Dispose of equipment and finish the injection procedure** |  |  |  |  |  |
|  | Discard the needle and syringe in the appropriate receptacle. | 0 |  | 2 |  | 4 |
|  | Remove gloves and dispose of them properly. | 0 |  | 2 |  | 4 |
|  | Tell the patient about finishing work and assist the patient to a position of comfort. |  |  |  |  |  |

Student’s name: Class: Date:

**Total score: /60 Converted score /10 Observer:**
