# Supplementary material for: Teacher-made models: the answer for medical skills training in developing countries?
Source: BMC Med Educ. 2012 Oct 19;12:98. doi: 10.1186/1472-6920-12-98 (PMC3533861; doi:10.1186/1472-6920-12-98)
Supplement: Additional file 3 — Compare the results of the pre-practice assessment. [file 1472-6920-12-98-S3.doc]

[**Appendix 3: Compare the results of the pre-practice assessment**](../Teacher%20made%20models%2024.07.doc)

Dependent Variable: total score of the pre-practicing assessment

Bonferroni

| (I) GROUP | (J) GROUP | Mean Difference  (I-J) | Std. Error | Sig. | 95% Confidence Interval | |
| --- | --- | --- | --- | --- | --- | --- |
|  |  |  |  |  | Lower Bound | Upper Bound |
| 1 | 2 | -.35119 | 1.72798 | 1.000 | -4.5379 | 3.8355 |
|  | 3 | -2.48328 | 1.73724 | .465 | -6.6924 | 1.7258 |
| 2 | 1 | .35119 | 1.72798 | 1.000 | -3.8355 | 4.5379 |
|  | 3 | -2.13209 | 1.74608 | .672 | -6.3626 | 2.0984 |
| 3 | 1 | 2.48328 | 1.73724 | .465 | -1.7258 | 6.6924 |
|  | 2 | 2.13209 | 1.74608 | .672 | -2.0984 | 6.3626 |
