# Supplementary material for: Teacher-made models: the answer for medical skills training in developing countries?
Source: BMC Med Educ. 2012 Oct 19;12:98. doi: 10.1186/1472-6920-12-98 (PMC3533861; doi:10.1186/1472-6920-12-98)
Supplement: Additional file 4 — Results of post-practice assessment using CM. [file 1472-6920-12-98-S4.doc]

Appendix 4: Results of post-practice assessment using CM

| No | CONTENTS | Group 1 | Group 2 | Group 3 |
| --- | --- | --- | --- | --- |
| 1 | Check right medication, wear mask and wash your hands. | 2.45 | 2.73 | 2.47 |
| 2 | Prepare medication. | 2.86 | 2.60 | 2.83 |
| 3 | Check the right client with the physician’s order, prepare patient. | 2.84 | 2.50 | 2.70 |
| 4 | Select appropriate injection site. | 2.80 | **2.48***** | **2.96**** |
| 5 | Cleanse the entry site. | 3.10 | 2.94 | 3.06 |
| 6 | Wash hands again with disinfection solution or alcohol swab. | 2.12 | 1.88 | 1.70 |
| 7 | Remove air bubbles in syringe. | 2.55 | 2.92 | 2.60 |
| 8 | Ensure that the bevel side of the needle is facing up. | 3.76 | 3.50 | 3.74 |
| 9 | Stretch the skin against the direction of insertion to the site. | 2.33 | 1.92 | 2.34 |
| 10 | Insert at 30-40 degree angle. | 2.82 | 2.46 | 2.68 |
| 11 | Advance the needle into the vein. | 2.82 | 2.60 | 2.55 |
| 12 | Check the right position of the needle. | 3.04 | 2.67 | 2.94 |
| 13 | Aspirate by pulling back gently on the plunger of syringe to determine the needle is in a blood vessel, release the tourniquet. | 3.76 | 3.33 | 3.83 |
| 14 | Inject the medication into the vein. | 2.39 | 2.62 | 2.79 |
| 15 | Dispose of equipment and finish the injection procedure | 3.16 | 2.88 | 2.98 |

** Significantly different from groups 2, p<0.05

*** Significantly different from groups 3, p<0.05
