# Supplementary material for: Teacher-made models: the answer for medical skills training in developing countries?
Source: BMC Med Educ. 2012 Oct 19;12:98. doi: 10.1186/1472-6920-12-98 (PMC3533861; doi:10.1186/1472-6920-12-98)
Supplement: Additional file 5 — Results of post-practice assessment using TM. [file 1472-6920-12-98-S5.doc]

**Appendix 5: Results of post-practice assessment using TM**

| No | CONTENTS | Group 1 | Group 2 | Group 3 |
| --- | --- | --- | --- | --- |
| 1 | Check right medication, wear mask and wash your hands. | 2.57 | 2.71 | 2.36 |
| 2 | Prepare medication. | 2.65 | **2.79***** | **2.45**** |
| 3 | Check the right client with the physician’s order, prepare patient. | **2.88***** | 3.17 | **3.34*** |
| 4 | Select appropriate injection site. | 2.90 | 2.67 | 2.79 |
| 5 | Cleanse the entry site. | 3.02 | 3.29 | 3.13 |
| 6 | Wash hands again with disinfection solution or alcohol swab. | 1.96 | 2.46 | 2.21 |
| 7 | Remove air bubbles in syringe. | 2.49 | 2.67 | 2.38 |
| 8 | Ensure that the bevel side of the needle is facing up. | 3.78 | 3.88 | 3.85 |
| 9 | Stretch the skin against the direction of insertion to the site. | 2.24 | 2.08 | 1.83 |
| 10 | Insert at 30-40 degree angle. | 2.57 | 2.42 | 2.70 |
| 11 | Advance the needle into the vein. | 2.41 | 2.13 | 2.30 |
| 12 | Check the right position of the needle. | 3.06 | 2.83 | 3.15 |
| 13 | Aspirate by pulling back gently on the plunger of syringe to determine the needle is in a blood vessel, release the tourniquet. | 3.51 | 3.67 | 3.74 |
| 14 | Inject the medication into the vein. | 2.65 | 2.46 | 2.60 |
| 15 | Dispose of equipment and finish the injection procedure | 2.88 | 2.77 | 2.55 |

* Significantly different from group 1, p<0.05

** Significantly different from group 2, p<0.05

*** Significantly different from group 3, p<0.05
