# Supplementary material for: Teacher-made models: the answer for medical skills training in developing countries?
Source: BMC Med Educ. 2012 Oct 19;12:98. doi: 10.1186/1472-6920-12-98 (PMC3533861; doi:10.1186/1472-6920-12-98)
Supplement: Additional file 6 — Results of assessment of performance on real patients. [file 1472-6920-12-98-S6.doc]

**Appendix 6: Results of assessment of performance on real patients**

| No | CONTENTS | Group 1 | Group 2 | Group 3 |
| --- | --- | --- | --- | --- |
| 1 | Check right medication, wear mask and wash your hands. | 3.55 | 3.31 | 3.32 |
| 2 | Prepare medication. | 3.16 | 2.98 | 3.21 |
| 3 | Check the right client with the physician’s order, prepare patient. | **2.73** - ***** | **3.29*** | **3.32*** |
| 4 | Select appropriate injection site. | 2.92 | 2.58 | 2.68 |
| 5 | Cleanse the entry site. | 2.69 | 2.96 | 3.00 |
| 6 | Wash hands again with disinfection solution or alcohol swab. | 2.29 | 2.13 | 2.04 |
| 7 | Remove air bubbles in syringe. | 2.98 | 2.77 | 2.79 |
| 8 | Ensure that the bevel side of the needle is facing up. | 3.82 | 2.71 | 3.90 |
| 9 | Stretch the skin against the direction of insertion to the site. | 2.37 | 2.10 | 2.21 |
| 10 | Insert at 30-40 degree angle. | 2.53 | 2.44 | 2.89 |
| 11 | Advance the needle into the vein. | 2.47 | 2.27 | 2.47 |
| 12 | Check the right position of the needle. | 2.86 | **2.56***** | **3.04**** |
| 13 | Aspirate by pulling back gently on the plunger of syringe to determine the needle is in a blood vessel, release the tourniquet. | 3.33 | 2.15 | 3.13 |
| 14 | Inject the medication into the vein. | 2.71 | 2.40 | 2.79 |
| 15 | Dispose of equipment and finish the injection procedure | 2.55 | 2.75 | 2.70 |

* Significantly different from group 1, p<0.05

** Significantly different from group 2, p<0.05

*** Significantly different from group 3, p<0.05
